# Metabolic sensitivity of pancreatic tumour cell apoptosis to glycogen phosphorylase inhibitor treatment

**DOI:** 10.1038/sj.bjc.6602243

**Published:** 2004-12-21

**Authors:** W-N P Lee, P Guo, S Lim, S Bassilian, S T Lee, J Boren, M Cascante, V L W Go, L G Boros

**Affiliations:** 1 Department of Pediatrics, Los Angeles Biomedical Research Institute at Harbor-UCLA Medical Center, RB1, 1124 West Carson Street, Torrance, CA 90502, USA; 2 SIDMAP, LLC, 10021 Cheviot Drive, Los Angeles, CA 90064, USA; 3 Department of Biochemistry and Molecular Biology, University of Barcelona, C/Marti I Franques 1, 08028 Barcelona, Spain; 4 UCLA Center for Human Nutrition, 900 Veteran Avenue, Los Angeles, CA, 90095, USA

**Keywords:** pentose cycle, ribose synthesis, glycolysis, apoptosis, stable isotope-based dynamic metabolic profiling (SIDMAP), inhibition of cell proliferation

## Abstract

Inhibitors of glycogen breakdown regulate glucose homeostasis by limiting glucose production in diabetes. Here we demonstrate that restrained glycogen breakdown also inhibits cancer cell proliferation and induces apoptosis through limiting glucose oxidation, as well as nucleic acid and *de novo* fatty acid synthesis. Increasing doses (50–100 *μ*M) of the glycogen phosphorylase inhibitor CP-320626 inhibited [1,2-^13^C_2_]glucose stable isotope substrate re-distribution among glycolysis, pentose and *de novo* fatty acid synthesis in MIA pancreatic adenocarcinoma cells. Limited oxidative pentose-phosphate synthesis, glucose contribution to acetyl CoA and *de novo* fatty acid synthesis closely correlated with decreased cell proliferation. The stable isotope-based dynamic metabolic profile of MIA cells indicated a significant dose-dependent decrease in macromolecule synthesis, which was detected at lower drug doses and before the appearance of apoptosis markers. Normal fibroblasts (CRL-1501) did not show morphological or metabolic signs of apoptosis likely due to their slow rate of growth and metabolic activity. This indicates that limiting carbon re-cycling and rapid substrate mobilisation from glycogen may be an effective and selective target site for new drug development in rapidly dividing cancer cells. In conclusion, pancreatic cancer cell growth arrest and death are closely associated with a characteristic decrease in glycogen breakdown and glucose carbon re-distribution towards RNA/DNA and fatty acids during CP-320626 treatment.

Tumour cell proliferation involves special metabolic adaptation to increased macromolecule synthesis, primarily increased synthesis of RNA, DNA and fatty acids, which triggers an increased need for glucose to be shunted into ribose-phosphate and acetyl-CoA precursor synthesis pathways. Entering frequently the ‘S’ phase of a tumour cell's life cycle requires the rapid breakdown of the intracellular glucose store glycogen, which is regulated by the brain (foetal) type glycogen phosphorylase (GP) enzyme complex (Shimada *et al*, 1999). Therefore, limiting glycogen breakdown is a strong signal for tumour cells of inadequate substrate glucose availability, which decelerates cycle progression and likely induces apoptosis. Numerous GP inhibitors (GPIs) have been synthesised targeting various substrate or cofactor binding sites, such as the AMP stimulatory, purine nucleoside and indole-binding sites (Hoover *et al*, 1998; Treadway *et al*, 2001). Inhibitors of the complex that bind to cyclin-dependent kinases (CDKs), such as flavopiridol, decelerate glycogen breakdown and are currently being evaluated in clinical trials for the treatment of gastrointestinal cancers (Kelland, 2000). 5-Chloro-1H-indole-2-carboxylic acid (1-(4-fluorobenzyl)-2-(4-hydroxypiperidin-1-yl)-2-oxoethyl) amide, CP-320626, was identified as a potent inhibitor of human liver glycogen phosphorylase-b (GP-b) with properties of selective and specific allosteric binding to the enzyme complex in close proximity to the AMP allosteric regulator and catalytic sites (Oikonomakos *et al*, 2000). The compound exerts its inhibitory effects by stabilising the less active T state of the GP complex, providing both a highly specific recognition site and clearly delineated mechanism of action that are of significant benefit in the search for improved GP inhibitors. CP-320626 bears more selective and effective binding properties than those of flavopiridol compounds, and the efficacy of its antiproliferative action and mechanisms involved in limiting human cancer growth are yet to be reported. In particular, whether and how inhibiting the GP complex with CP-320626 effectively limits substrate utilisation for macromolecule synthesis within the highly activated glucose metabolic network of tumour cells is of great interest.

In this paper, we report significant changes in glucose carbon re-distribution among major metabolic pathways for macromolecule synthesis in pancreatic adenocarcinoma cells treated with CP-320626, using the [1,2-^13^C_2_]glucose stable isotope-based dynamic metabolic profiling (SIDMAP) approach to track and quantify such changes using previously established methods (Boros *et al*, 2003; Green, 2003). The observed metabolic changes are correlated with the induction and appearance of cell cycle arrest and apoptosis-related molecular markers. Our data reveal that the metabolic profile characteristic of limited synthesis from glucose of nucleotide macromolecule precursors and fatty acids precedes the appearance of cell cycle arrest and apoptosis-related molecular markers following inhibition of GP by CP-320626. These results provide new information about the pathways and mechanisms through which inhibition of glycogen breakdown results in insufficient macromolecule synthesis to support proliferation, consistent with the metabolic hypothesis of cell growth regulation and apoptosis formation in pancreatic cancer (Boros *et al*, 2002a, 2002b).

## MATERIALS AND METHODS

The tracer for this metabolic profiling study, stable isotope [1,2-^13^C_2_]-D-glucose, was purchased with >99% purity and 99% isotope enrichment for each position from Cambridge Isotope Laboratories, Inc. (Andover, MA, USA). CP-320626 was kindly provided by Pfizer Inc. (Groton, Connecticut) for the study.

### Cells and cell culture

Human pancreatic ductal adenocarcinoma MIA PaCa-2 cells (American Type Culture Collection (ATCC) CRL1420) and control CRL-1501 human fibroblasts were purchased from ATCC. MIA PaCa-2 cells have an average doubling time of 40 h in DMEM with 10% foetal bovine serum and 2.5% horse serum (Gibco/BRL, Gaitersburg, MD, USA) in the presence of antibiotics. The cells were incubated at 37°C, 5% CO_2_ and 95% humidity, and passed by using trypsin 0.25% (Gibco/BRL) no more than 10 times after receipt from the ATCC and prior to use in this study. MIA cells have previously been used in both *in vitro* and *in vivo* experiments and responded to treatments with pentose cycle inhibitors and glycolysis enzyme inhibitors with characteristic metabolic profile changes showing restricted macromolecule synthesis, which correlated with decreased proliferation with great specificity (Boros *et al*, 1997). CRL-1501 human immortalised fibroblasts were used in this study to compare normal and tumour cell sensitivities to GP inhibitor treatment in an escalating regime of drug treatment from 25 to 100 *μ*M CP-320626.

Confluent cultures (75%) of MIA or CRL-1501 cells were incubated in [1,2-^13^C_2_]-D-glucose-containing media (100 mg dl^−1^ total concentration=5 mM; 50% isotope enrichment – that is, half unlabelled glucose, half labelled with the stable isotope ^13^C tracer). Cells were plated at a density of 10^6^ per T75 culture flask and CP-320626 added in a concentration range of 25*–*100 *μ*M, dissolved in 10% DMSO – 90% culture media. Control cultures were treated with vehicle only. The doses of CP-320626 for the present study were selected based on *in vitro* experiments demonstrating that this drug effectively controls GP activity in the presence or absence of glucose in human cells in the 10–100 *μ*M dose range (Andersen and Westergaard, 2002). In separate experiments, MIA cells were treated with graded doses of 2-deoxy-D-glucose (2-DOG) to compare the efficacy of the antiproliferative effects of CP-320626 to the antiproliferative effects of 2-DOG, an established glycolysis inhibitory substrate. Glucose and lactate levels in the medium were measured using a Cobas Mira chemistry analyzer (Roche Diagnostics, Pleasanton, CA, USA).

### RNA ribose stable isotope studies

RNA ribose was isolated by acid hydrolysis of cellular RNA after Trizol purification of cell extracts. Total RNA amounts were assessed by spectrophotometric determination, in triplicate cultures. Ribose was derivatised to its aldonitrile acetate form using hydroxylamine in pyridine with acetic anhydride (Supelco, Bellefonte, PA, USA) before mass spectral analyses. We monitored the ion cluster around *m/z* 256 (carbons 1–5 of ribose; chemical ionisation (CI)) and *m/z* 217 (carbons 3–5 of ribose) and *m/z* 242 (carbons 1–4 of ribose; electron impact ionisation (EI)) to determine molar enrichment and the positional distribution of ^13^C in ribose. By convention, the base mass of ^12^C-compounds (with their derivatisation agents) is given as *m*_0_, as measured by mass spectrometry as described elsewhere (Boros *et al*, 2002a, 2002b). Ribose molecules labelled with a single ^13^C atom on the first carbon position (*m*_1_) recovered from RNA were used to gauge the ribose fraction produced by direct oxidation of glucose through the G6PD pathway. Ribose molecules labelled with ^13^C on the first two carbon positions (*m*_2_) were used to measure the fraction produced by transketolase. Doubly labelled ribose molecules (*m*_2_ and *m*_4_) on the fourth and fifth carbon positions were used to measure the molar fraction produced by triose phosphate isomerase and transketolase.

### Lactate

Lactate from the cell culture media (0.2 ml) was extracted by ethylene chloride after acidification with HCl. Lactate was derivatised to its propylamine-heptafluorobutyrate ester form and the *m/z* 328 (carbons 1–3 of lactate; CI) was monitored for the detection of *m*_1_ (recycled lactate through the PC) and *m*_2_ (lactate produced by the Embden–Meyerhof–Parnas pathway) for the estimation of pentose cycle activity (Lee *et al*, 1998a). In this study, we recorded the *m*_1_/*m*_2_ ratios in lactate produced and released by MIA pancreatic adenocarcinoma cells in order to determine pentose cycle activity *vs* anaerobic glycolysis in response to CP-320626 treatment.

### Glutamate

Glutamate label distribution from glucose is suitable for determining glucose oxidation *vs* anabolic glucose use within the TCA cycle, also known as anaplerotic flux. Tissue culture medium was first treated with 6% perchloric acid and the supernatant was passed through a 3 cm^3^ Dowex-50 (H+) column. Amino acids were eluted with 15 ml 2 N ammonium hydroxide. To further separate glutamate from glutamine, the amino-acid mixture was passed through a 3 cm^3^ Dowex-1 (acetate) column, and then collected with 15 ml 0.5 N acetic acid. The glutamate fraction from the culture medium was converted to its trifluoroacetyl butyl ester (TAB). Under EI conditions, ionisation of TAB-glutamate produces two fragments, *m/z* 198 and 152, corresponding to C2–C5 and C2–C4 of glutamate (Lee *et al*, 1996). Glutamate labelled on the 4–5 carbon positions indicates pyruvate dehydrogenase activity, while glutamate labelled on the 2–3 carbon positions indicates pyruvate carboxylase activity for the entry of glucose carbons to the TCA cycle. TCA cycle anabolic glucose utilisation is calculated based on the *m*_1_/*m*_2_ ratios of glutamate (Leimer *et al*, 1977).

### Fatty acids

Palmitate, stearate, cholesterol and oleate were extracted after saponification of cell pellets in 30% KOH and 100% ethanol using petroleum ether. Fatty acids were converted to their methylated derivative using 0.5 N methanolic-HCl. Palmitate, stearate and oleate were monitored at *m/z* 270, 298 and 264, respectively, with the enrichment of ^13^C-labelled acetyl units, which reflect synthesis, elongation and desaturation of the new lipid fraction as determined by mass isotopomer distribution analysis (MIDA) of different isotopomers (Lee *et al*, 1995, 1998b).

### Gas chromatography/mass spectrometry (GC/MS)

Mass spectral data were obtained on the HP5973 mass-selective detector connected to an HP6890 gas chromatograph. The settings were as follows: GC inlet 250°C, transfer line 280°C, MS source 230°C, MS Quad 150°C. An HP-5 capillary column (30 m length, 250 *μ*m diameter, 0.25 *μ*m film thickness) was used for glucose, ribose and lactate analyses. As transketolase has the highest metabolic control coefficient in the nonoxidative branch of the pentose cycle (Sabate *et al*, 1995; Comin-Anduix *et al*, 2001), we use the term *transketolase* throughout the paper. It should be noted, though, that transketolase and transaldolase, besides other enzymes, are all participants in nonoxidative pentose cycle metabolism in human cells.

### *In vitro* cell proliferation assay

The *in vitro* testing was accomplished by seeding MIA PaCa-2 cells into 96 well (Falcon 3072, Franklin Lakes, NJ, USA) flat bottom proliferation plates (5000 cells well^−1^). On the second day, the culture media were replaced by 2% FBS-DMEM (minimum growth media). Three full plates were assigned either to 50 or 100 *μ*M CP-320626 treatment regimens as follows: cells in the first column (eight wells) on each plate were used as controls without treatment, and the second through 12th columns were treated with the compounds under testing. One extra plate without treatment was used to characterise the natural growth patterns of MIA cells on the 96-well plates during the study period, and it was used to correct for differences occurring due to culturing conditions other than experimental interventions. Promega (Madison, WI) MTS *in vitro* cell viability/proliferation assay was performed to investigate cell density and cell viability in response to increasing concentrations of CP-320626. Treatments were discontinued 24 h prior to the proliferation assay by replacing the culture media with 50 *μ*l minimum growth DMEM. The assay was performed by adding 50 *μ*l of tetrazolium dye solution to 50 *μ*l of cell culture media for 4 h. Then the stop solution was added (de-ionised sterile water, 100 *μ*l) and the formazan product of the tetrazolium salt was measured by a spectrophotometer at 490 nm.

### TUNEL apoptosis assay

The terminal deoxynucleotidyl transferase (TdT)-biotinylated 16-deoxy-UTP nick end labelling (TUNEL) apoptosis assay allows the recognition of apoptotic nuclei in cell preparations fixed on slides by Fragment End Labelling (FragEL) of DNA. MIA cells were fixed at 5 × 10^6^ cells ml^−1^ density in 1% paraformaldehyde in PBS, at pH 7.4 for 10 min at room temperature. In all, 100 *μ*l of the cell suspension from the trypsinised culture was dried on a silanised microscope slide, and then quenched in 3.0% hydrogen peroxide in PBS for 5 min at room temperature. Samples were rinsed twice with PBS for 5 min each in a Coplin jar before treatment with working strength TdT enzyme, as described in the ApopTag *In Situ* Apoptosis Detection protocol and Kit (Intergen Co., Purchase, NY, USA).

### Data analysis and statistical methods

Each experiment was carried out using triplicate cell cultures for each condition within each experiment and experiments were repeated once. Mass spectroscopic analyses were carried out by three independent automatic injections of 1 *μ*l samples by the automatic sampler, and accepted only if the standard sample deviation was less than 1% of the normalised peak intensity. Statistical analysis was performed using the Student's *t*-test for unpaired samples. Two-tailed significance at the 99% confidence interval (*μ*±2.58*σ*), *P*<0.01, indicated significant differences in glucose carbon metabolism in control and CP-320626-treated MIA cells, as shown below.

## RESULTS

The stable isotope-based dynamic metabolic profiling (SIDMAP) approach has been successful in determining pentose cycle activity, glycolysis, the contribution of the two branches of the pentose cycle to nucleic acid synthesis, *de novo* fatty acid synthesis and acetyl-CoA glucose enrichment in HepG2 liver and MIA pancreatic adenocarcinoma cells, as described previously (Boros *et al*, 1997, 2002a 2002b). The MIA cell line showed a close correlation between limited cell proliferation and decreased [1,2-^13^C_2_]glucose substrate flow towards RNA/DNA and fatty acid synthesis in negative and positive control drug testing metabolic profiling studies (Boren *et al*, 2001). Using the same approach for the present study, we incubated human pancreatic MIA adenocarcinoma cells with [1,2-^13^C_2_]glucose in the presence or absence of CP-320626 in order to determine ^13^C-label distribution in RNA ribose, lactate and glutamate, and to determine the contribution of glucose carbons to *de novo* nucleic and fatty acid synthesis, glycolysis and TCA cycle anaplerotic flux. Based on the number of ^13^C-labelled carbons present in the extracted small molecular weight intermediary metabolites, the ratio of metabolite synthesis via each pathway was determined.

**Figure 1:**
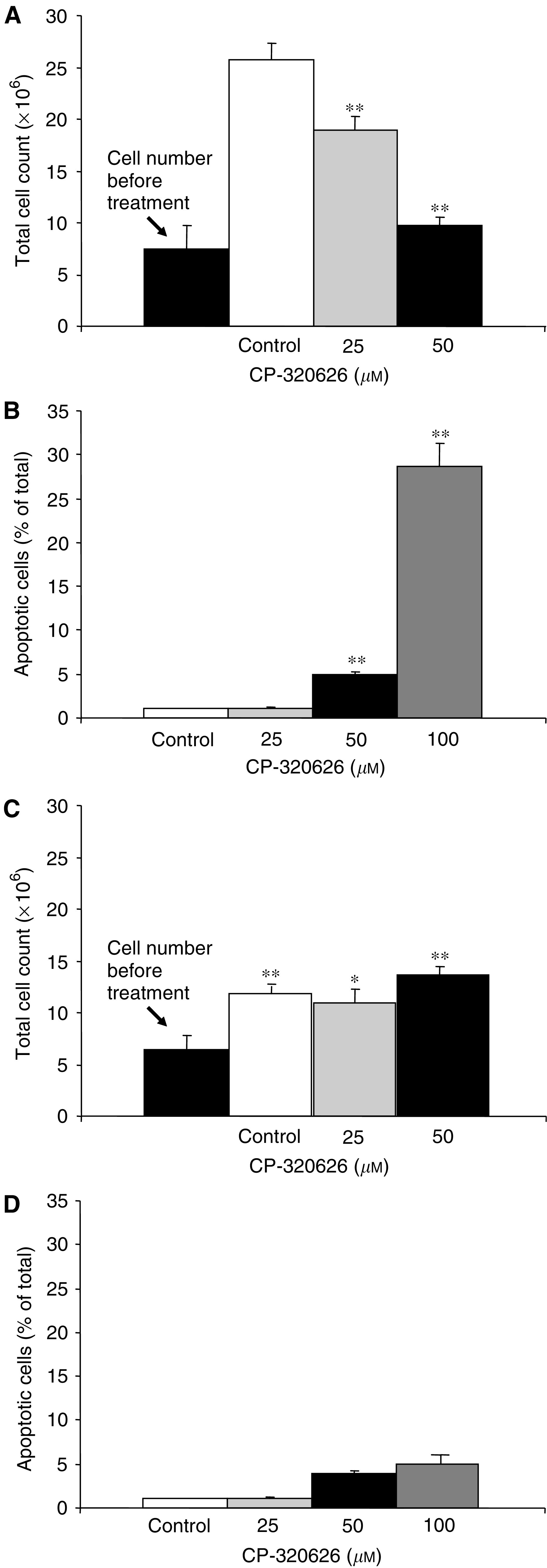
(**A**) Cell count before and after 72 h dose-escalating CP-320626 treatment of MIA pancreatic adenocarcinoma cells in culture. Cell numbers before the study and 72 h later in response to 50 *μ*M CP-320626 treatment are not statistically different from the starting cell number. Control cells were grown for 72 h with vehicle only. (**B**) TUNEL assay indicates 4% apoptotic MIA cells formation in 50 *μ*M CP-320626-treated cultures, which increases about seven-fold, to 28%, after 100 *μ*M CP-320626 treatment. (**C**) Cell count before and after 72 h dose-escalating CP-320626 treatment of CRL-1501 human fibroblasts. (**D**) TUNEL assay indicates only 1–5% apoptotic CRL-1501 cell formation in 50 *μ*M CP-320626-treated cultures. (*n*=6; mean+s.d.; ^*^P<0.05, ^**^P<0.01).

The optical cell count of MIA pancreatic adenocarcinoma cultures using a haemocytometer before and after 72 h of dose-escalating CP-320626 treatment indicated a dose-dependent inhibition of cell growth in MIA cultures (Figure 1A

). CP-320626 completely inhibited cell proliferation as compared to the starting cell number at the beginning of the *in vitro* cell proliferation study; however, it was not clear to us whether this dose-dependent inhibition of cell growth was attributable to limited cell proliferation or to increased cell death. Evaluation of the cultures using a phase-contrast microscope revealed only a few floating (separated or dead) cells, which points to limited cell growth due to cell cycle arrest. The TUNEL assay indicated a dose-dependent appreciable increase in apoptosis induction in MIA cell cultures after treatment with either 50 or 100 *μ*M CP-320626. The frequency of apoptotic cells increased from 4 to 28%, respectively (Figure 1B). CRL-1501 human fibroblasts failed to exhibit growth retardation or apoptosis in proliferation and TUNEL assays using 25, 50, 75 or 100 *μ*M CP-320626 (Figure 1C and D).

**Figure 2:**
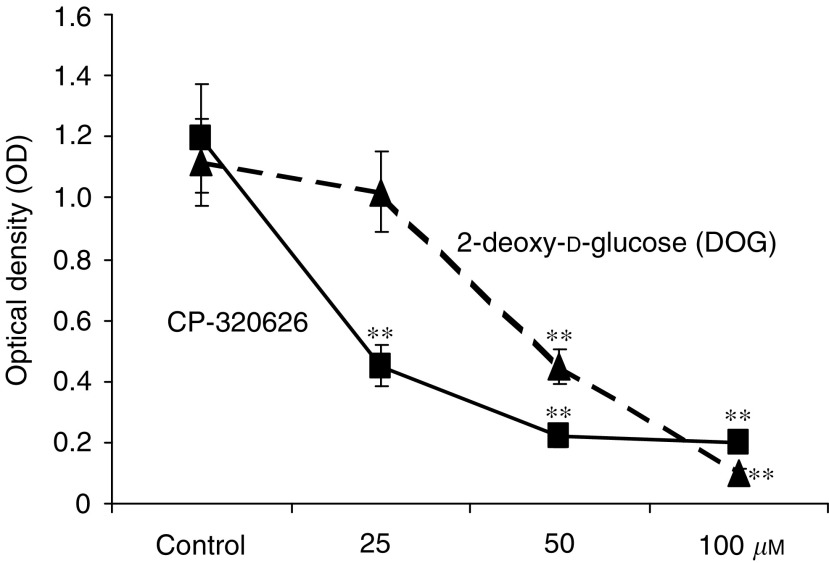
Optical density (OD) of MIA cultures after 72 h dose-escalating CP-320626 treatment in the MTS cell proliferation/survival assay. This test indicates significantly fewer MIA cells with deprived oxidative metabolism after increasing doses of CP-320626 treatment. This experiment was performed in comparison with 2-deoxy-D-glucose, which is a well-known growth-inhibitory substrate through cell cycle arrest and apoptosis (*n*=8; mean±s.d.; ^*^P<0.05, ^**^P<0.01).

Optical densitometry (OD) of the cultures also indicated a dose-dependent rapid decrease in cell proliferation as revealed by the MTS assay (Figure 2

). CP-320626 and 2-DOG-treated cultures showed similar growth characteristics with significant inhibition of cell proliferation. The glucose metabolite 2-DOG swiftly causes cycle arrest and apoptosis in tumour cells due to a characteristic decrease in glycolysis and pentose cycle substrate flow (Aft *et al*, 2002); however, CP-320626 was more effective in inhibiting proliferation and oxidative metabolism, as indicated by the sharp decrease of MTS staining of treated MIA cultures with GP inhibitor drug.

Cell number-adjusted glucose consumption and lactate release indicated no significant changes in cellular glucose consumption or lactate release into the culture media after 25 or 50 *μ*M CP-320626 treatment, signifying that MIA cells responding to this GP inhibitor drug well maintain both glucose uptake and viability. The average glucose consumption was 16 ng 24 h^−1^ 10^−6^ MIA cells and the average lactate production was 11 nM 24 h^−1^ 10^−6^ cells, with no significant change after treatment with CP-320626.

**Figure 3:**
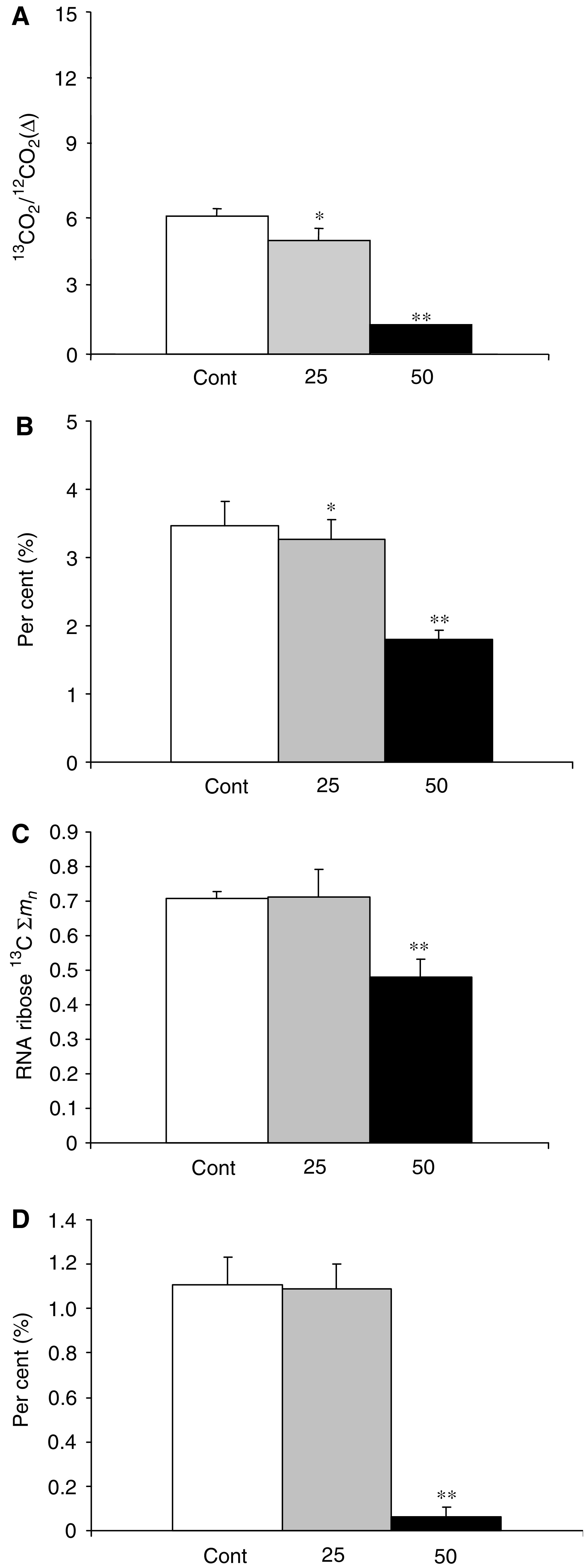
SIDMAP of MIA cells after treatment with increasing doses of the glycogen phosphorylase inhibitor CP-320626 indicates severe changes in intracellular glucose carbon distribution. Carbon dioxide release from glucose is decreased, as demonstrated by a fall in ^13^C/^12^C ratios in panel **A**; the decrease in pentose cycle direct glucose oxidation and recycling into lactate is shown in panel **B** as per cent (%) of glycolysis; the decrease in nucleic acid (RNA) ribose ^13^C molar enrichment is shown in panel **C** as ^13^C ∑*m*_*n*_, and the severe decrease in TCA cycle anabolic glucose use (anaplerosis) is shown in panel **D** as per cent (%) of glucose oxidation within the cycle (*n*=6; mean+s.d.; ^*^P<0.05, ^**^P<0.01).

**Figure 4:**
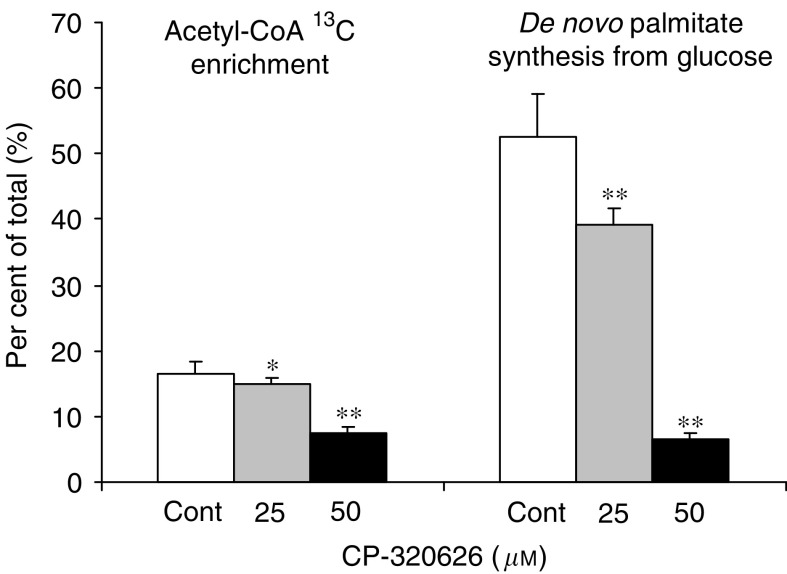
Continued SIDMAP reflecting decreased fatty acid synthesis in MIA cells after treatment with increasing doses of the glycogen phosphorylase inhibitor CP-320626. In control cultures, about 18% of acetyl units and about 52% of intracellular palmitate arise from glucose, expressed as per cent (%) of the total. CP-320626 treatment decreased both acetyl unit synthesis and *de novo* fatty acid palmitate synthesis from glucose in a dose-dependent fashion (*n*=6; mean+s.d.; ^*^P<0.05, ^**^P<0.01).

However, we observed discernible significant changes in intracellular glucose carbon re-distribution among major glucose-utilising macromolecule synthesis pathways in response to CP-320626 treatment. Figure 3

reveals the [1,2-^13^C_2_]glucose stable isotope–based dynamic metabolic profile (SIDMAP) of MIA cells. The SIDMAP panel indicates that 25 and 50 *μ*M CP-320626 treatment decreases CO_2_ release from glucose significantly (Figure 3; panel A), and that there is a dose-dependent 7.8 and 49.6% decrease in direct glucose oxidation in the pentose cycle via G6PDH and its recycling into lactate through transketolase and transaldolase (Figure 3; panel B). It is also revealed from the SIDMAP that there is a 33% decrease in nucleic acid (RNA) ^13^C enrichment (Figure 3; panel C) and a 94.4% decrease in TCA cycle glucose anaplerosis after 50 *μ*M CP-320626 treatment (Figure 3; panel D). The continued metabolic profile is also consistent with an 8.7 and 56.2% decrease in acetyl-CoA glucose ^13^C enrichment, and a 26.6 and 88.4% decrease in *de novo* fatty acid palmitate synthesis from glucose after 25 and 50 *μ*M CP-320626 treatment, respectively (Figure 4

). On the other hand, CRL-1501 cells exhibited no ^13^C tracer accumulation into palmitate, stearate or oleate, indicating the lack of *de novo* fatty acid synthesis from glucose and less dependence on rapid glycogen turnover for survival and growth.

The metabolic profile and apoptosis formation in control human CRL-1501 fibroblast cell line cultures were not affected by GP inhibitor treatment at doses identical to that of MIA cell cultures. Apoptosis formation was observed but remained between 1 and 5% in all control and CP-320626-treated CRL-1501 cultures (Figure 1; panels C and D).

## DISCUSSION

Glycogen phosphorylation is the first step in glycogenolysis, which releases glucose from glycogen as glucose-1-phosphate (G-1-P). Other than its contribution to maintaining blood glucose homeostasis, little is known of G-1-P's role in cell proliferation. Since G-1-P is part of the hexose phosphate moieties, it is an immediate precursor for many downstream reactions within the glucose metabolic network. These include glycolysis, pentose synthesis, energy generation and fatty acid synthesis. Our experimental data prove that, even though glycogen is not the direct precursor of pentose production, it indirectly affects pentose synthesis and fatty acid synthesis rates in addition to affecting other distal metabolic reactions. The impact of GP inhibition on these distal metabolic reactions is significant and extensive. We observed significant reversions in the extent of glucose utilisation for ribose and fatty acid synthesis even at the lowest doses of CP-320626 well before significant increase in cell cycle arrest or apoptosis induction occurred.

When the GP inhibitor CP-320626 hinders glucose metabolic pathways, MIA cells experience restricted ability to proliferate in culture although they maintain metabolic activities as indicated by ^13^C tracer accumulation into all studied metabolites. They continue consuming glucose and producing lactate after both 25 and 50 *μ*M treatments with CP-320626. These changes are consistent with continued cell viability, but lack of proliferation that is expressive of cell cycle arrest. However, further increase (doubling) in drug concentration induces a seven-fold enhancement of apoptosis. Tumour cells heavily rely on all available extra- and intracellular glucose sources for the rapid synthesis of nucleotides, amino acids and fatty acids, which is demonstrated by the depleted glycogen pool of transformed cells (Hughes, 1976; Karnik *et al*, 1981; Bismut *et al*, 1995). It is evident from the metabolic profile reported here that the ability of MIA cells to progress in the cell cycle is severely impeded by limited glycogen breakdown in the presence of CP-320626 and that the cell cycle arrest readily progresses into apoptosis with higher doses of the drug. As the chemical structure and binding characteristics of CP-320626 are several-fold more effective and are more specific to the GP enzyme protein than those of flavopiridol (Hoover *et al*, 1998), it is evident that the antiproliferative action of CP-320626 is mediated through direct metabolic substrate flow changes and not through the interruption of the cyclin-dependent kinase pathway (Kelland, 2000). It has been documented that GP A and B activities are increased in the muscle of diabetic pancreatic cancer patients (Liu *et al*, 2000). Therefore, therapies of pancreatic cancer using specific GP inhibitors may improve host glucose status and insulin sensitivity via improving skeletal muscle glycogen synthesis and glycogen storage, while inhibiting tumour cell proliferation and glucose cycling through glycogen.

Metabolic profile changes reported herein are followed by the appearance of molecular markers for apoptosis, pointing to the fact that if tumour cells experience interruption of the altered metabolic network that turns glycogen rapidly into nucleic acid ribose and fatty acids, that interruption will impose a blockage on cell cycle progression. The reported significant alterations in substrate re-distribution and macromolecule synthesis indicate that regulators of key metabolic processes of substrate utilisation play important roles in the induction of apoptosis in MIA pancreatic adenocarcinoma cells. It is likely that this scenario exists in all rapidly dividing human malignant cells.

**Figure 5:**
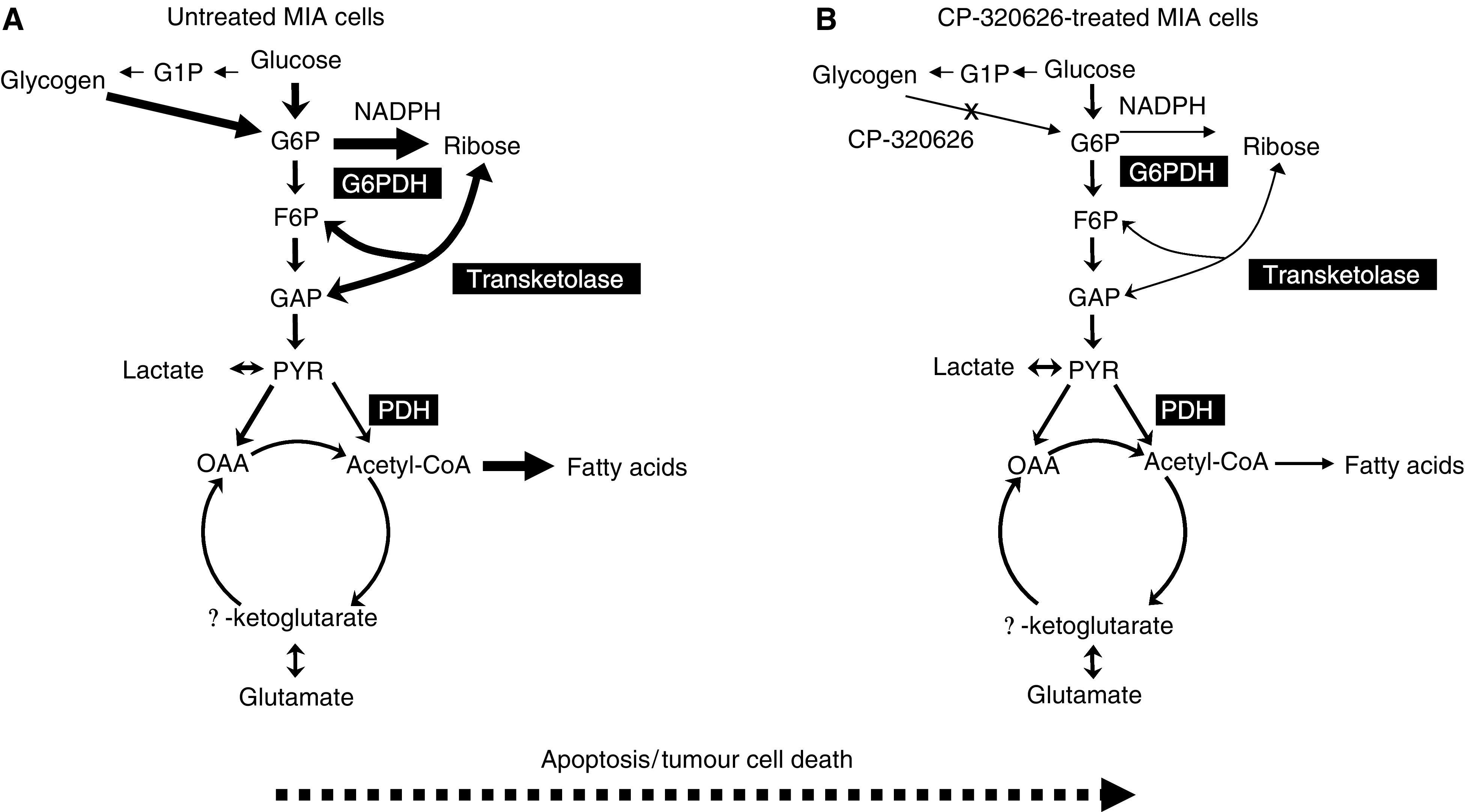
Metabolic profile changes associated with glycogen phosphorylase inhibitor treatment in MIA pancreatic adenocarcinoma cells. MIA cells in the absence of CP-320626 utilise glucose as the major substrate for *de novo* nucleic acid and fatty acid synthesis as shown to the left (**A**). CP-3206262 limits glycogen breakdown as indicated on the panel to the right (**B**). As a result, tumour cells become less capable of synthesising nucleic acid ribose and fatty acids, which significantly limits their cycle progression and growth. One interesting feature of this metabolic profile is that it appears at lower concentrations of CP-320626 and precedes apoptosis formation. (**G6P**: glucose -6-P; **G1P**: glucose-1-P; **F6P**: fructose-6-P; **GAP**: glyceraldehyde-3-P; **PYR**: pyruvate; OAA: oxaloacetate).

It is evident that SIDMAP studies combined with molecular techniques are remarkably valuable tools in revealing metabolic mechanisms that result in cell cycle arrest and apoptosis. Human tumour cells readily become wholesale re-distributors of glucose and they primarily utilise this substrate for macromolecule synthesis. The malignant phenotype's inherent biochemical mandate to effectively compete for and preferentially obtain all glucose available to it makes glucose tracers optimal tools for SIDMAP studies, as shown consistently herein. Such studies provide a precise elucidation of key molecular mechanisms responsible for cell cycle events and apoptosis, thereby suggesting which specific metabolic enzyme inhibitors are likely to have significant potential as antiproliferative agents. The metabolic changes in CP-320626-treated MIA cells are consistent with decreased synthesis of key precursor intermediates participating in macromolecule synthesis, DNA/RNA formation and membrane assembly (Figure 5

). The prominent decrease observed in the contribution of glucose to nucleic acid ribose synthesis indicates that glycogen and its rapid breakdown into glucose are required for promoting tumour cell proliferation. In turn, the lack of adequate glycogen breakdown and glucose cycling ensures that apoptosis will ensue. This observation may identify several new enzymatic target sites in the apoptotic pathway of mammalian cells, which is regulated primarily through a metabolic balance between glycolysis, the pentose cycle and glycogen synthesis/breakdown. The fact that apoptosis formation remained less than 5% in all control and CP-320626-treated CRL-1501 human fibroblast cultures indicates that a significant diversity exists between transformed and nontransformed cells in their sensitivity to inhibiting rapid glycogen breakdown as a substrate for the synthesis of cell proliferation-related macromolecules, primarily RNA and fatty acids. For example, CRL-1501 human fibroblasts do not utilise glucose carbons for *de novo* fatty acid synthesis in the presence of more than 2% foetal bovine serum in the culture medium. This metabolic diversity by the differential use of glucose in CRL-1501 fibroblasts and MIA cells is important to understand tumour cells' sensitivity to GP inhibitor treatment.

These findings suggest that defective ribose and fatty acid synthesis are the likely causes of cell cycle arrest and apoptosis in cultured MIA pancreatic adenocarcinoma cells in response to GP inhibitor treatment, and provide a strong rationale for the use of a GP inhibitor as an adjunct for the treatment of human cancer in clinical trials. Finally, the observation that inhibition of glycogen phosphorylation induces cell cycle arrest and apoptosis strongly supports the metabolic hypothesis that the regulation of cell growth and cell death in human cancers occurs through the substrate level regulation of macromolecule synthesis, which determines cell cycle progression and apoptosis formation.
